# The Terror Attacks of 9/11 and Suicides in Germany: A Time Series Analysis

**DOI:** 10.1097/MD.0000000000003228

**Published:** 2016-04-18

**Authors:** Daniel Medenwald

**Affiliations:** From the Institute of Medical Epidemiology, Biostatistics and Informatics, Martin-Luther-University Halle-Wittenberg, Halle/Saale, Germany.

## Abstract

Data on the effect of the September 11, 2001 (9/11) terror attacks on suicide rates remain inconclusive. Reportedly, even people located far from the attack site have considerable potential for personalizing the events that occurred on 9/11. Durkheim's theory states that suicides decrease during wartime; thus, a decline in suicides might have been expected after 9/11.

We conducted a time series analysis of 164,136 officially recorded suicides in Germany between 1995 and 2009 using the algorithm introduced by Box and Jenkins.

Compared with the average death rate, we observed no relevant change in the suicide rate of either sex after 9/11. Our estimates of an excess of suicides approached the null effect value on and within a 7-day period after 9/11, which also held when subsamples of deaths in urban or rural settings were examined.

No evidence of Durkheim's theory attributable to the 9/11attacks was found in this sample.

## INTRODUCTION

The terror attacks of September 11, 2001 (9/11) shook people worldwide because they disclosed the immediate threat of terrorism for the United States and its allies.

Subsequently, an enormous amount of media attention, particularly on television, played a key role in transporting the images and information regarding the attacks in New York to distant regions. It was later revealed that the consumption of 9/11-related television images was positively associated with posttraumatic stress disorder (PTSD) symptoms.^1,2^

Symptoms of distress were found not only in people in the regions closest to the attack sites, but also in those located far from the attack sites,^3,4^ which indicates that there was no relevant proximity effect for PTSD. Accordingly, data from US residents who were indirectly exposed to the attacks showed a similar prevalence of PTSD as those who were directly exposed.^3^ Therefore, the 9/11 terror attacks could have had a profound impact on mental health worldwide. Addressing a more profound level of the effects of such events, it was soon emphasized that there is a considerable possibility that the 9/11 events were personalized by people located far from New York.^5,6^ Indeed, the perceived similarity between oneself versus victims is the major factor that determines the internalization of such events.^5^ Even on the highest political and governmental levels in Germany, unconditional solidarity (“Uneingeschränkte Solidarität”) was declared by Chancellor Schröder to the people of the United States. The experienced vulnerability of the western world led to major changes in everyday life in Germany, such as an increased need for security, making the situations in Germany and the United States appear similar. Thus, social relations in Germany might have favored an increased predisposition for solidarity and equivalently augmented social cohesion, when, although it was not immediately experienced, an external threat was perceived.

A century ago, Durkheim published his theory of declining suicides occurring at times of increased social cohesion, such as during wartime.^7^ Applying Durkheim's theory to the 9/11 terror attacks, we would expect lower suicide rates in the aftermath of 9/11. Although the suicide rate after 9/11 has been assessed by several studies, the data from non-American populations are still scant and often inconclusive. One study from the Netherlands found evidence for increased suicide rates after 9/11,^5^ whereas data from England indicated a decrease in suicides during the time period surrounding 9/11.^8^ In the latter study, the suicide rates had already started to decline on September 9, thus weakening the evidence for causal interference from the terror attacks. A study in the United States, which confirmed a decrease in the number of suicidal deaths, and also a subordinated proximity effect,^9^ had methodical shortcomings.^10^

Furthermore, the investigation of a country that was not directly affected by the attacks enables the examination of the differences in suicides between urban and rural areas, as hypothesized by Durkheim, without the confounding effect of the distance to the attack sites, which must be taken into account in American studies.

Reflecting on previous findings, we aimed to assess the effect of 9/11 on the suicide rate in Germany.

## METHODS

### Data Acquisition

The data were obtained from the microdata set of official statistics provided for scientific use by the Research Data Centres (RDCs) of the Federal Statistical Office and the Statistical Offices of the Länder. This anonymized data set includes all the suicidal deaths registered daily in Germany from 1995 to 2009—a total of 164,136 suicides. The cause of death is defined as the underlying disease that initiated the causal chain leading to death. The data access and analysis took place via “remote execution.” An ethical approval was not necessary because only secondary data were used. Results were only released after a data security check was performed by the RDC.

### Statistical Analysis

**FIGURE 1:**
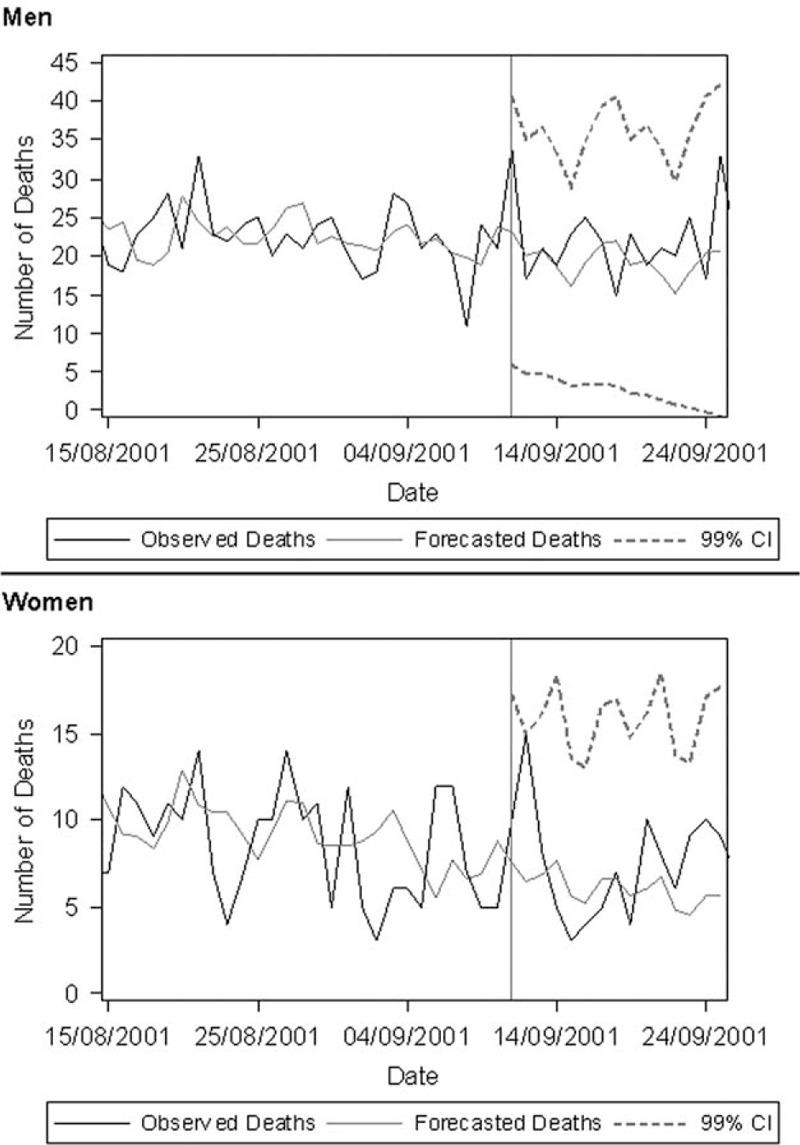
Observed and forecast daily suicide rates from 8/15/01 to 9/15//01 in Germany. The forecast was computed using the PROC FORECAST procedure in SAS with a first-order autoregressive model.

As a first step to assess the changes in the suicide rate related to the terror attacks, the number of expected deaths was forecast for a period after 9/11. To accomplish this, we used the PROC FORECAST procedure in SAS and applied a first-order autoregressive model (Figure 1).^11^

Time series analyses and, more specifically, seasonal autoregressive integrated moving average (SARIMA) models were used for further statistical analyses. The models and their respective effects were estimated separately for men and women. Model identification was based on the algorithm introduced by Box and Jenkins.^12,13^ To estimate the excess in suicidal deaths on 9/11 and subsequent days, a transfer function was added to the SARIMA model. The transfer function we used is a binary time series (pulse function) that is set to “1” either solely on 9/11, or on 9/11 and the ensuing days, with the latter modeling a persisting effect up to 7 days after 9/11. Thus, the number of suicidal deaths can be represented by the following formula: 
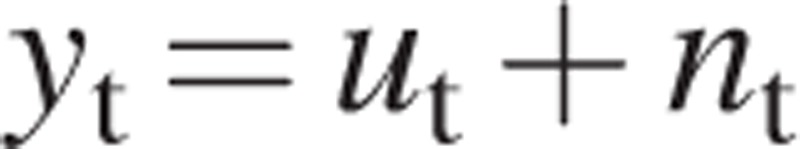


where *y*_t_ is the output series of suicidal deaths, *u*_t_ represents the portion that can be explained by the transfer function, and *n*_t_ is an error process. The results from the time series analyses are given as the excess in deaths with corresponding 99% confidence intervals (CIs).

Due to the data privacy regulations of the RDCs, we replaced less than 4 suicides per day with random numbers from the estimated binominal distribution in Figure 1; however, all the statistical analyses were conducted with the full sample. For multiple testing, a *P* of less than 0.01 (rather than 0.05) was assumed to be statistically significant.

Additionally, separate analyses were conducted for the urban (major cities with more than 100,000 inhabitants) and remaining (rural) areas.^14^

To account for age and sex, we computed the age and sex-standardized suicidal mortality rate (per 100,000 inhabitants per day) for each day between September 7 and September 19 of 2000, 2001, and 2002, using direct standardization with the new European standard population. The daily mortality rates of 2000 and 2002 were compared with those of 2001 by taking their respective differences into account. The sum of variances of the respective days was used for statistical evaluation, implementing the assumption that the yearly mortality rates are independent. The adjusted variance was computed as the weighted average of the age and sex-specific variances.

All the statistical analyses and data management were performed using SAS V.9.3 (SAS, Cary, NC).

## RESULTS

On average, 21.9 (99% CI 21.7, 22.1) suicides were recorded for men, and 8.0 (99% CI 7.9, 8.1) suicides were recorded for women. As per the Box–Jenkins approach, a periodicity of 7 days was modeled in both sexes to obtain residuals that were not significantly auto-correlated, which hints at a weekly cycle of suicidal deaths.

As Figure 1 shows, the observed number of suicides on and after 9/11 did not exceed the 99% CI of the forecast. From visual assessment, there might be a specific peak on 9/11 in the male suicide rate, which was, when modeled as a pulse function (Table 1), slightly lower than expected (the negative excess can be attributed to the steep rise preceding 9/11) and not relevantly in excess of random death fluctuations. Finally, when considered as pulse functions (up to a duration of 7 days), there was no enduring effect influencing the suicidal deaths of either sex over the time spans beginning on 9/11 (Table 1). This is also supported by wide confidence limits and becomes particularly obvious when the estimated excesses are compared with the average number of suicides per day.

**TABLE 1:**
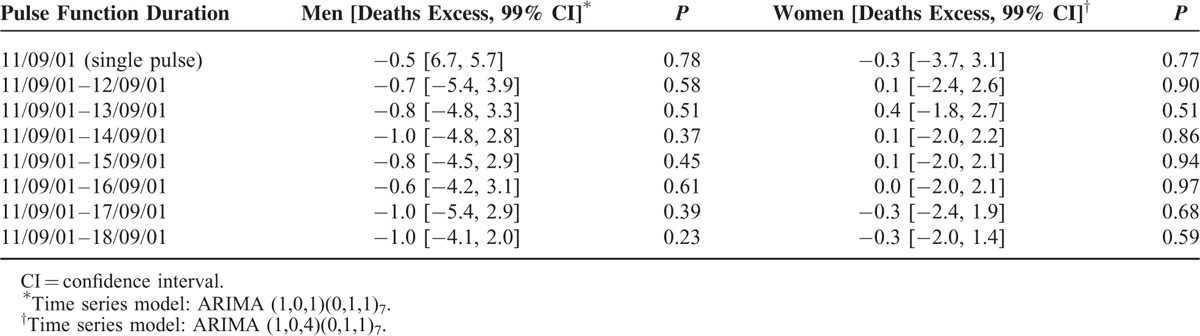
Estimated Excess in Suicidal Death on and After 9/11 in Germany From 1995 to 2009

When the suicides were stratified into urban and rural populations, the excess of suicidal deaths on and during the period after 9/11 approached the null effect value with wide CIs (Table 2).

**TABLE 2:**
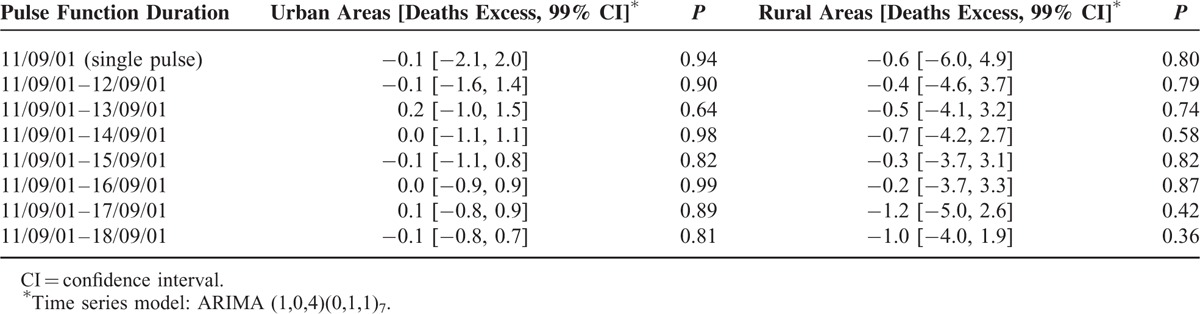
Estimated Excess in Suicidal Death on and After 9/11 in Germany from 1995 to 2009 in Urban and Rural Areas

**FIGURE 2:**
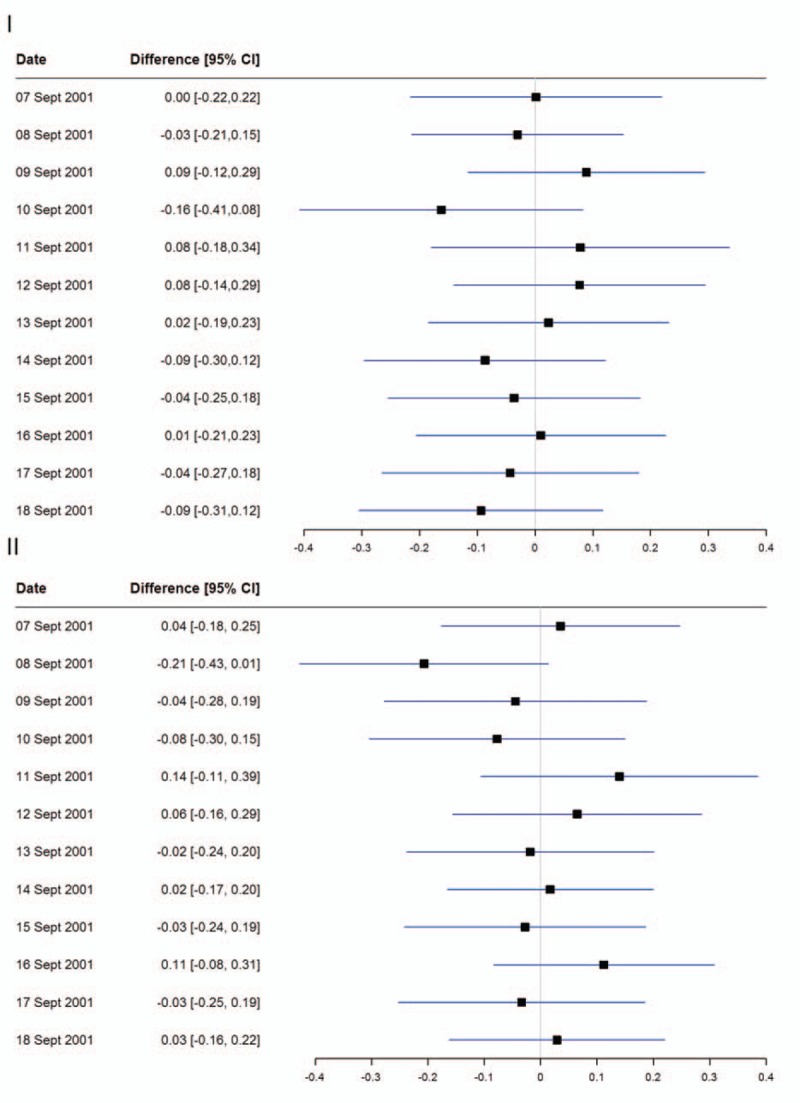
Forest plot of the difference in daily suicide rates between 2001 and 2000 (I)/2002 (II). The forest plot shows the effect estimates as absolute differences after age and sex standardization using the new European standard population.

Comparing mortality on a daily basis between September 7 and 18, 2001, with the mortality rate in 2000 and 2002 (Figure 2), we again found differences approaching the null effect value accompanied by wide CIs. Interestingly, a slight numerical rise in mortality was observed on September 11, 2001, particularly when compared with mortality in 2002; however, the CIs still included 0.

## DISCUSSION

Summarizing our findings over a 15-year period of deaths, we disclosed no relevant change in the suicide rates in Germany attributable to 9/11. This contradicts previous reports of increasing^5^ and decreasing suicide rates^8^ in European countries. Thus, with respect to 9/11, we found no evidence of causal interference according to Durkheim's theory. One possible explanation for our results is that the long time period we used enabled us to more accurately assess daily fluctuations in suicide numbers. This is important, as the consideration of singular segments of a time series might easily bias results towards an underestimation of the error term.

Another explanation for our finding might be related to regional distance; however, previous research proved the existence of a 9/11 mental health effect even in distant^3^ or not directly affected regions.^4^ Nevertheless, the strongest effect is expected when people regard themselves to be either similar to the victims or in a similar situation as the victims.^15^ People in Germany might have perceived the events of 9/11 to be too personally remote to influence their suicidal behavior, even with the presence of a certain degree of mental distress.

Although we observed no rise in suicide rates, we cannot rule out an alteration in the number of suicide attempts during the relevant time period. Nevertheless, according to Durkheim's theory, both the number of attempts and the vigor with which they are committed should decrease due to wars or war-like acts. In contrast, it was reported that suicide attempts rose in the United States in the aftermath of 9/11.^16^

In a comprehensive review, Kushner and Sterk^17^ challenged Durkheim's theory and typology and emphasized that socioeconomic status predicts health, including mental well-being, better than the concept of social cohesion, as even the definition of “social cohesion” is not universally accepted. The same authors^17^ pointed to the shortcomings of Durkheim's theorem. They emphasized the “antiurban” bias that lies in the concept of “social cohesion,” and also the omission of suicide attempts by women in Durkheim's analyses, which could have considerably altered his inferences. They also mentioned other factors such as reciprocity and trust, which can be considered to be an extension of the formal idea of mere “social cohesion.”

Such reasoning might well apply to the findings of the present study, as values have potentially shifted from family (the traditional corner stone of social cohesion) to socioeconomic factors in Germany, and the social mobility of today differs from that of Durkheim's time. Even Durkheim's concept of women being socially integrated in a stronger way than men seems questionable in today's societies.

In Durkheim's day, the impact of (social) media and the internet on suicides was unknown; however, they currently play an important role in society with the potential to alter public opinion or be altered by it.^18–22^ Also, the current highly integrated financial system, which was affected considerably by the events of 9/11, might have caused antagonistic effects to a decreasing suicide rate in the aftermath of war by a normative deregulation, leading eventually to a rise in suicides.^23^ It is extremely difficult, if not impossible, to disentangle the actual 9/11 effect and the related external factors in opposition to it.

An ecological study's primary limitation lies in its inability to draw inferences on an individual level, which is difficult with time series data. The inaccuracy of death certificates has been previously reported,^24^ but was not expected to cause bias in our case, as the assignments of cause of death were unlikely to have differed systematically as 9/11 approached.

A social cohesion gain in socially disintegrative areas, as described by Durkheim, would give strong support for his ideas; however, there was no change in the suicide rate in urban areas in Germany on or after 9/11.

In conclusion, no relevant effect of the 9/11 terror attacks was found on the suicide rates in Germany, and no evidence underpinning Durkheim's theory was found in this nationwide sample.
